# Differential Expression of Sphingosine-1-Phosphate Receptors in Abdominal Aortic Aneurysms

**DOI:** 10.1155/2012/643609

**Published:** 2012-03-26

**Authors:** Z. Qu, Bernice L. Y. Cheuk, Stephen W. K. Cheng

**Affiliations:** Division of Vascular Surgery, Department of Surgery, The University of Hong Kong, Hong Kong

## Abstract

*Objective*. Inflammation plays a key role in the pathophysiology of abdominal aortic aneurysms (AAAs). Newly discovered Sphingosine-1-Phosphate Receptors (S1P receptors) are critical in modulating inflammatory response via prostaglandin production. The aim of the current study was to investigate the expression of different S1P receptors in AAAs and compared with normal aortas at the protein level. *Materials and Methods*. Aortic specimens were harvested during aortic reconstructive surgery for the AAA group or during organ transplant for the control group. The protein expression of S1P1, 2 and 3 in AAAs and normal aortas was assessed by Western blotting and immunohistochemical analysis. *Results*. There were 40 AAAs and 20 control aortas collected for the receptor analysis. For Western blot analysis, S1P1 expression was not detected in either group; S1P2 protein was constitutively detected in both types of aortas but its expression level was significantly decreased by 73% (*P* < 0.05) in AAAs compared with the control group. In contrast, strong S1P3 expression was detected in AAAs aortas but not in normal aortas. Immumohistochemical staining showed similar results, except a weak S1P3 signal was detectable in normal aortas. *Conclusions*. Western blot and staining results consistently showed the down-regulation of the S1P2 protein with simultaneous up-regulation of the S1P3 protein in AAAs. Since those newly discovered receptors play an important role in the inflammatory cascade, the modulating of S1P signaling, particularly via S1P2 and S1P3, could represent novel therapeutic targets in future AAA treatments.

## 1. Introduction

Abdominal aortic aneurysm (AAA) is the localized dilation of the infrarenal aorta. If surgical treatment is not applicable, an AAA progresses to rupture with a high mortality rate and causes 1%–3% of elderly male deaths in developed countries each year [1]. Analysis of the aneurysmal wall has demonstrated that connective tissue degradation, increased atherosclerosis, and chronic inflammation are the common pathological features of AAAs [1–3]. 

Sphingosine-1-phosphate (S1P) is a newly discovered low-molecular-weight zwitterionic lysophospholipid molecule that is generated from the metabolism of sphingomyelin by a series of enzymes including sphingosine kinase, S1P phosphatase, and S1P lyase in mammals [4]. The main sources of S1P are platelet cells in plasma, while other cell types such as erythrocytes, neutrophils, and mononuclear cells can also produce and release S1P upon activation [4, 5]. S1P exerts a wide range of physiological activities, particularly inflammatory reactions through the interactions with five different receptor subtypes 1, 2, 3, 4, and 5. They are the members of the endothelial differentiation gene family of G protein-coupled receptors [4], and differential expressions of S1P receptors are thought to modulate the cellular inflammatory response [6]. A precise S1P/S1P receptor balance is found to be responsible for the signaling of cell growth and regulation of cell metabolism in mammal [7, 8]. An imbalance of this system also participates in pathologic conditions such as cancer and inflammatory diseases [9, 10]. S1P2 is the major expressed S1P receptor, while S1P1 or S1P3 is only weakly expressed in healthy vascular endothelial cells [11] and vascular smooth muscle cells (VSMCs) [12]. Consistently with this receptor multiplicity and pleiotropic signaling mechanisms, S1P receptors influence numerous cell functions. Particularly, differential expressions of S1P receptors have been demonstrated to either promote or inhibit the inflammatory infiltration in diverse cell types by inducing cyclooxygenase 2 (COX-2) expression [6] with subsequent prostaglandin E_2_ (PGE_2_) or prostacyclin (PGI_2_) production [13–15]. Our previous study showed that inflammatory mediators such as Cox-2 and prostaglandin E_2_ are also widely expressed in AAA explants [16]. Those phenomena implicating S1P receptors may play some roles in the pathogenesis of AAA.

S1P1, S1P2, and S1P3 receptors are the major S1P receptor subtypes in the vascular system [11, 12]. However, the expressions of these three S1P receptors in AAA remain unknown. In the present study, we aimed to investigate the S1P1, 2 and 3 receptor protein expressions in AAAs and compare them with healthy aortas.

## 2. Materials and Methods

### 2.1. Human Abdominal Aorta Tissues Collection

Cross-sections of aneurysm wall, which were dissected of luminal thrombus, were removed from the AAA patients who underwent open surgical aneurysmal repair in a local hospital. Control aortic tissues were obtained from the corresponding location of healthy organ donors without known cardiovascular diseases and connective tissue disorders during the transplant operation. Aneurysmal patients with the Marfan syndrome and other connective tissue disorders were excluded from this study.

Each collected specimen was thoroughly washed with normal saline solution, and then the tunica intima elimination procedure was conducted using a scalpel and forceps. All specimens were divided into two parts for the western blotting which was kept in a −80°C freezer and immunohistochemical analysis, respectively.

All experiments were performed with the approval from the local institution's ethics committee. Informed consent was obtained from AAA patients and organ donors' relatives.

### 2.2. Western Blot Analysis

Frozen tissues were first thawed and then lysed with cell lysis buffer (Cell Signaling. Technology, Danvers, MA, USA) containing protease inhibitor (Roche, Basel, Switzerland). The protein concentration of each specimen was measured based on the Bradford method utilizing the Bio-Rad Protein Assay Kit (Bio-Rad Laboratories, Hercules, CA, USA) with bovine serum albumin (BSA) as the standard. After the protein denaturing procedure with loading buffer (pH 6.8 24 mM Tris-HCl, 684 mM glycerol, 14 mM SDS, 142 mM beta-mercaptoethanol, 0.3 mM bromophenol blue), each sample (50 *μ*g) was resolved on 12% SDS-polyacrylamide gel electrophoresis (PAGE) gel (Bio-Rad Laboratories) at room temperature then transferred onto a polyvinylidene fluoride (PVDF) membrane (Bio-Rad Laboratories) at 4°C. After blocking in 10% TBS–0.01% Tween 20 (TBST) diluting nonfatty milk (Bio-Rad Laboratories) for two hours at room temperature, the membranes were then incubated overnight at 4°C with primary antibodies against S1P1 receptor (Catalogue no. sc-48356, dilution 1 : 100, Santa Cruz Biotechnology, Santa Cruz, CA, USA) or S1P2 receptor (Catalogue no. sc-25491, dilution 1 : 200, Santa Cruz Biotechnology) or S1P3 receptor (Catalogue no. sc-30024, dilution 1 : 100, Santa Cruz Biotechnology) with GAPDH (1 : 1000, Cell Signaling Technology) as the positive control. After membrane washing using TBST solution, HRP-conjugated goat anti-mouse (1 : 2000) or goat anti-rabbit (1 : 4000) secondary antibody (Dako, Glostrup, Denmark) was added and the membranes were then incubated for 1 hour at room temperature. After washing, signals were visualized by luminol reagents (Bio-Rad Laboratories) and the densitometry of each exposing blotting was analyzed by ImageJ 1.44 software (National Institutes of Health, Bethesda, MD, USA). The relative expression of the studied receptors' protein was calculated by the detected signal divided by the internal positive control (GAPDH) expression signal in each sample.

### 2.3. Immunohistochemical Study

The staining procedure was performed on paraffin-embedded aortic tissue (5 *μ*m) sections according to the manufacturer's instructions (DakoCytomation EnVision + System-HRP (DAB) Kit (Dako)). Specificity of S1P receptor antibodies was firstly validated by positive and negative tests using healthy adult rat brain paraffin sections. Briefly, all sections were antigen retrieved with boiling sodium citrate buffer (pH 6) and incubated with either mouse anti-S1P1 receptor antibody (1 : 25), rabbit anti-S1P2 receptor antibody (1 : 100), or rabbit anti-S1P3 receptor antibody (1 : 100) (Santa Cruz Biotechnology) overnight at 4°C. After staining, all specimens were subjected to the dehydration procedure and sealed for microscopy observation.

To avoid staining underestimation due to considerable regional variations, 5 continuous × 200 microscopy views of each stained specimen, which had the largest amount of positive stained VSMCs, were captured and recorded (Nikon, Tokyo, Japan). Two researchers scored the positive immunostaining using the scoring system according to the Wang and colleagues study [17]. Briefly, a proportion subscore from 0 to 4 (i.e., 0—0% positive stained, 1—1%–25% positive stained, 2—26%–50% positive stained, 3—51%–75% positive stained, 4—76%–100% positive stained) and an intensity subscore from 0 to 3 (i.e., 0: no staining, 1: weak staining, 2: moderate staining, 3: intense staining) were first assigned by each observer for each slide. A weighted score was then determined by multiplying the proportion subscore and the intensity subscore. Finally, a mean value of the five weighted scores for each specimen was calculated.

### 2.4. Statistical Analysis

All data were expressed as means ± SD. Statistical analysis was performed by SPSS 18.0 software (SPSS, Chicago, IL, USA). Any statistical differences between the two groups' experimental results were determined by independent sample *t*-tests. Correction for ages and sex of patients on receptor expression levels were made using a linear model. A *P* value < 0.05 was considered as statistically significant.

## 3. Results

### 3.1. Patient Characteristics

There were 40 AAA specimens and 20 control aortas obtained from the corresponding surgical patients and organ donors. Most of patients were male, and the control patients were younger than the AAA patients. Patient characteristics are listed in Table 1.

### 3.2. Western Blot Analysis

**Figure 1:**
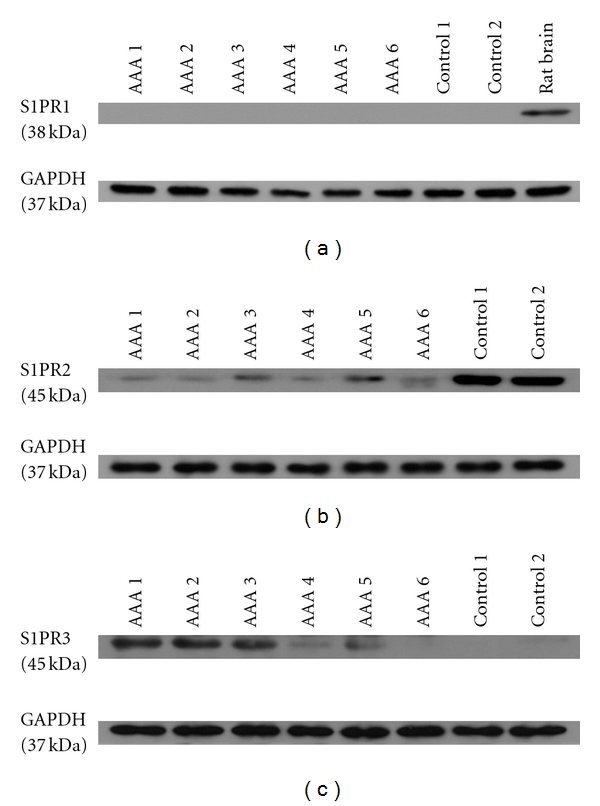
Representative pictures of western blot analysis of S1P receptors in AAA and control aortic tissues. S1P1 expression was undetectable in both AAA and normal aortas, while it became detectable in rat brain tissues (a). Expression level of S1P2 protein was significantly decreased in AAAs compared with control aortas (b). S1P3 expression was detectable in AAAs only (c). GAPDH antibody was used as internal positive control in each WB experiment.

S1P1 receptor protein (38 kDa) was undetectable in both tissues (Figure 1(a)). For S1P2 receptor protein (45 kDa), positive signals were detected in both AAA and control aortic tissues, with AAA tissues had a significantly lower protein expression level compared with control aortas (Figure 1(b)). In contrast, S1P3 receptor protein (45 kDa) was highly expressed in AAA aortas but was undetectable in control aortas (Figure 1(c)).

**Figure 2:**
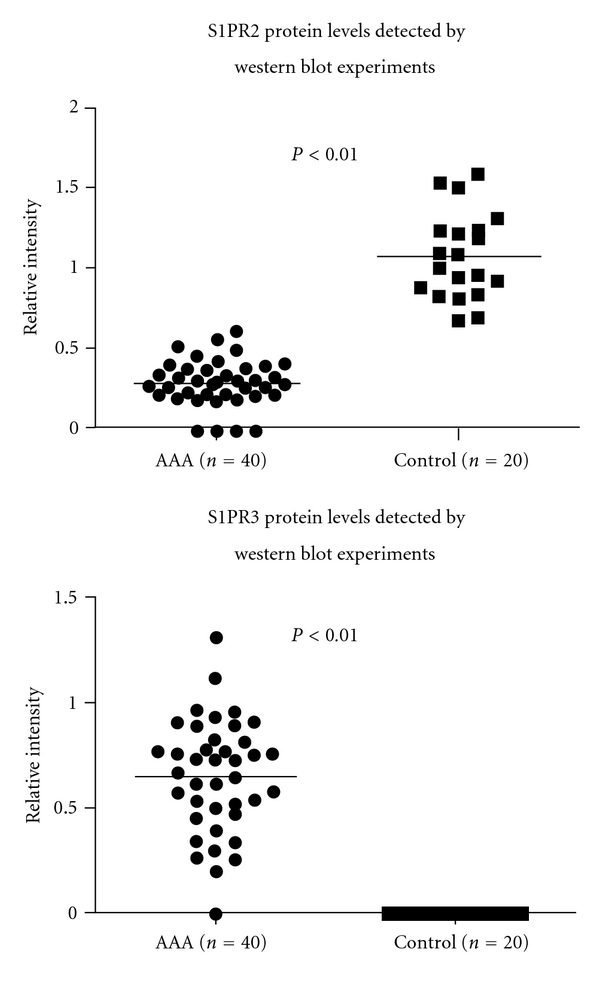
Quantitative analysis of S1P2 (upper panel) and 3 (lower panel) protein expression levels in western blot analysis. The relative S1P2 protein level was decreased by 73% (*P* < 0.05) in AAAs (relative expression intensity of 0.29) compared with normal aortas (relative expression intensity of 1.08). S1PR3 protein levels were significantly upregulated in AAA tissues with average relative intensity of 0.65, whereas it was undetectable in normal aortas.

The relative intensities of S1P1 and 2 receptors expression by western blot analysis are shown in Figure 2 upper and lower panels, respectively. The protein level of S1P2 receptor was decreased by 73% (*P* < 0.05) in the AAA tissues (mean relative intensity of 0.29) compared with the control aortic tissues (mean relative intensity of 1.08). S1PR3 protein levels were significantly upregulated in AAA tissues with average relative intensity of 0.65, whereas it was undetectable in normal aortas. 

### 3.3. Immunohistochemical Staining Analysis

**Figure 3:**
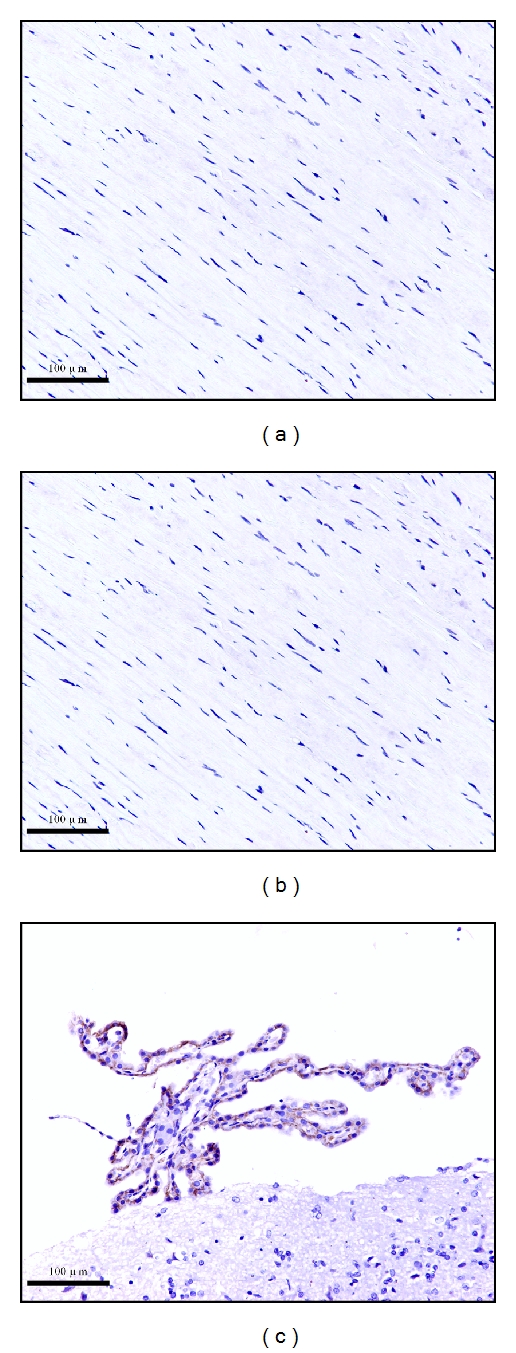
Representative staining pictures of S1P1 protein in AAA (a), control aortic (b) sections, and healthy adult rat brain paraffin sections (c) (×200). S1P1 protein was undetectable in both types of tissues.

**Figure 4:**
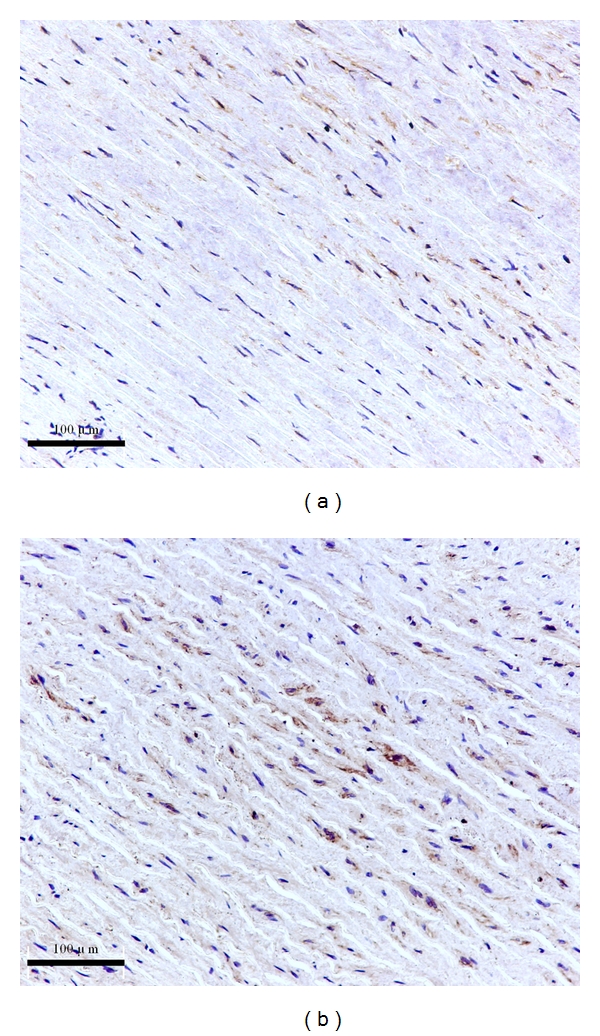
Representative staining pictures of S1P2 in AAAs and control aortic sections (×200). S1P2 protein was more pronounced in the normal aortas (b) than that in the AAAs (a).

**Figure 5:**
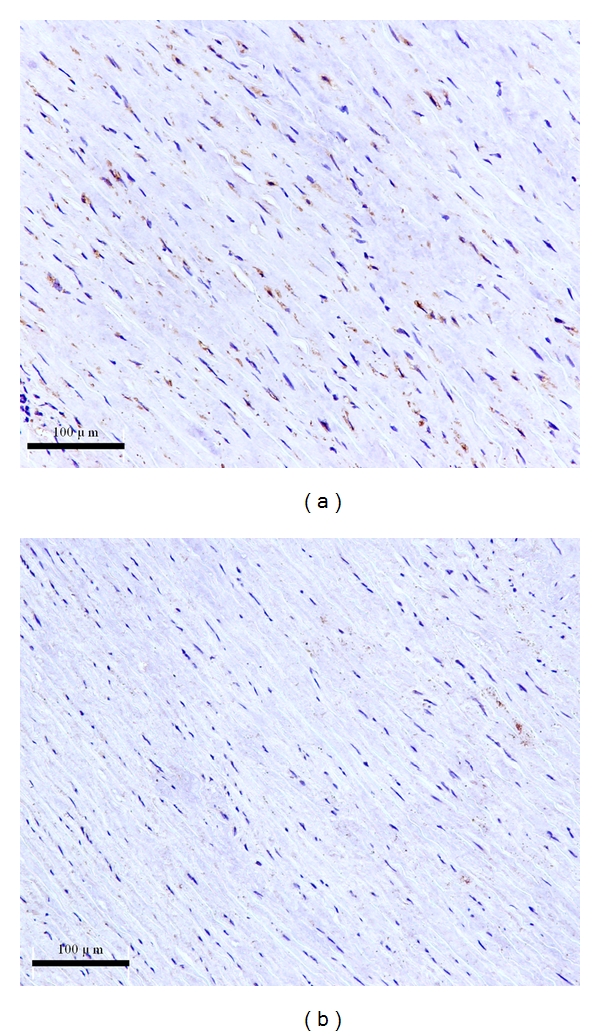
Representative staining pictures of S1P3 in AAA and control aortic sections (×200). More S1P3 protein was found in the AAA (a) than that in the normal aortas, in which it was almost undetectable.

S1P1 receptor expression level was undetectable in both AAA (a) and normal aortas (b), as shown in Figure 3. A Positive control of S1P1 receptor was performed in healthy adult rat brain for validating its specificity (Figure 3(c)). S1P2 receptor protein was expressed in both types of aortas, with pronounced S1P2 receptor staining observed in control aortas (Figure 4). S1P3 receptor protein was found in the AAA tissues (Figure 5(a)), but it was almost undetectable in normal aortas (Figure 5(b)). The staining scores of both types of tissues sections are shown in Table 2.

Positive staining of S1P2 and S1P3 receptors in the aortic walls showed that they were localized in VSMC plasma membrane and cytoplasm but absent in the nucleus (Figures 4 and 5).

### 3.4. Correction for Ages and Sex

Giving the age and sex discrepancy existed in the patients, corrections for ages and sex of the receptor protein expressions was made. The association between S1P2 and S1P3 protein (both IHC and WB expression levels) in the patients were robust to correction in a linear model with age and sex (*P* < 0.008).

## 4. Discussion

Among the studied S1P receptors, only S1P2 and S1P3 receptor proteins were differentially expressed in AAA tissues compared with the control aortas, while S1P1 receptor protein was absent in both types of tissues. Differential S1P receptors expressions have been shown to participate in diverse physiological processes, such as cell survival and apoptosis [18], and pathological processes, such as angiogenesis, inflammation, cancerogenesis, and immune regulation [9]. Inflammation is one of the common pathological features of AAAs [3]. Thus, the present novel findings may implicate the importance of these receptors in the inflammation attribute to AAA pathogenesis. Nevertheless, atherosclerosis is indeed regarded as a chronic inflammatory disease with atherosclerotic plaques containing inflammatory infiltrates, which implicated in the formation of AAA [19]. Thus, the possibility of S1P receptor expression related to the atherosclerotic event cannot be excluded.

S1P1 receptor is undetectable in both types of aortic tissues. Other researchers found that only some specific cell types, such as endothelial cells, cardiomyocytes, neural stem cells, as well as B cells, and T cells, express marked S1P1 [4]. Healthy rat adults' VSMCs express S1P1 receptor weakly [20], and the deletion of S1P1 receptor is embryonically lethal since it causes the failure of dorsal migration of VSMCs to form the tunica media layer of arteries [21], suggesting that S1P1 receptor should be critical in vascular development rather than in maintaining VSMCs metabolism [21]. A more recent study suggested that S1P1 is involved in the phenotype regulation of adult smooth muscle cells [22]. They utilized a rat carotid artery balloon injury model and demonstrated that there was a transient over expression and activation of S1P1 receptor after injury. This action can facilitate VSMCs transfer into the proliferative and migratory phenotype. However, such S1P1 receptor over expression will be restored to the basal value by 7 days after injury, suggesting that this irritable S1P1 receptor activation may be a short-term injury response. Thus, we postulated that the S1P1 receptor protein may be transiently increased during early AAA lesion, but its high expression will subsequently return to the basal level or become undetectable at the advanced stage of AAA development. In addition, S1P1 receptor is possibly responsible for the development of circulation system and expressed in the endothelial cells, rather than expressed in smooth muscle cells in mature aortas [21] which may explain for absent of S1P1 levels in the late stages of aneurysm.

S1P2 receptor protein was detected in both types of aortic tissues, particularly in control aortas. Indeed, S1P2 receptor has been previously shown to be the major S1P receptor expressing population in a wide variety of tissues in humans, like vascular endothelial [11] and smooth muscle cells [12], but not in inflammatory infiltrates [9]. This particular receptor can facilitate VSMCs' contractile phenotype expressions and negatively regulate their proliferation and migration [22]. Moreover, S1P2 receptor is capable of inducing Cox-2 expression and producing prostacyclin (PGI_2_) in response to exogenous S1P stimulus [15, 23, 24]. PGI_2_ possesses anti-inflammatory functions and simultaneously relaxes VSMCs and suppresses their proliferation and migration [25]. Thus, the decreased expression of anti-inflammatory S1P2 receptor in VSMCs of AAAs, and probably not expressed in the inflammatory infiltrates, may impair the production of PGI_2_ and ultimately lead to the pronounced inflammation response in AAA patients [16]. Therefore, the S1P2 receptor downregulation of VSMCs is obviously an important etiological factor in AAA development.

Markedly S1P3 receptor protein was found in AAA tissues. S1P3 receptor possesses a promoting inflammatory response property as it can induce Cox-2 expression and concomitant PGE_2_ production in various cell types [13, 14, 26, 27]. As a pronounced inflammatory infiltrate, PGE_2_ was also found in AAA explants in our laboratory previously [28] though the extent of its involvement in vascular inflammation is still unclear. In addition, a very recent study suggested that S1P3 mediates the chemotactic effect of its ligand-S1P in macrophages *in vitro* and *in vivo*, which plays a crucial role in atherosclerosis by promoting inflammatory monocyte/macrophage recruitment and altering smooth muscle cell behavior [29]. We suggested that the S1P3 receptor protein may be critical in the strengthened inflammatory response and thus atheroslcerosis via the chemotactic property and the PGE_2_ pathway during AAA development. However, the causal relationship between the inflammatory cells recruitment and the prostaglandins needed to be further explored.

In the present study, a weak S1P3 staining signal was found in normal aortas, though it became undetectable using western blot analysis. This staining result was consistent with Ryu et al. study that S1P3 is found to be weakly expressed in human healthy VSMCs [12].

Though age and sex discrepancy existed in the studied patients, the present analysis found that the expression of the S1P2 and 3 receptor proteins are not age and sex dependent. Thus, the up- or downregulated receptor probably related to the inflammatory cascade underlying the AAA pathogenesis but is not simply a feature of aging or sex difference.

The limitation of the present study is only an observational analysis on surgical aneurysmal aortas. Thus, we cannot extrapolate the observations to the initiation or promotion of aneurysm formation. Nevertheless, the present work has provided evidence that aneurysmal aortic tissue exhibits a decreased activity of S1P2 and enhanced S1P3 receptor, which may contribute to the inflammation of aortic walls involved in AAA pathology.

Our findings of the differential expression of S1P receptors in AAA compared with normal aortas are novel and may be helpful to delineate the important inflammatory mechanisms in AAA development. This investigation has provided a new concept in the inflammatory response in the lesions, and the regulation of S1P receptor via S1P2 and S1P3 may open a new regime for AAA treatment in the future.

## Figures and Tables

**Table 1:** Patient characteristics of AAA patients group and control group.

	AAA group (*n* = 40)	Control group (*n* = 20)
Age (mean age ± SD)	70.08 ± 9.72 yrs	42.05 ± 13.55 yrs
Gender (male %)	95%	40%
Smoking (%)	67.5%	—
Hypertension (%)	65%	5%
Diabetes (%)	7.5%	0%
Cardiac disease (%)	45%	0%
Renal disease (%)	27.5%	5%

**Table 2:** Staining scores of immunohistochemical analysis of S1P receptors in both types of tissues.

	AAA group (*n* = 40)	Control group (*n* = 20)
	Mean staining score (range)	
S1P1 receptor	—	—
S1P2 receptor	2.4 (0.6–4.8)	8.3 (5.2–10.8)
S1P3 receptor	5.2 (0.6–10.6)	0.25 (0–0.8)
